# Numerosities and Other Magnitudes in the Brains: A Comparative View

**DOI:** 10.3389/fpsyg.2021.641994

**Published:** 2021-04-15

**Authors:** Elena Lorenzi, Matilde Perrino, Giorgio Vallortigara

**Affiliations:** Centre for Mind/Brain Science, CIMeC, University of Trento, Rovereto, Italy

**Keywords:** brain, numerosity, number, comparative cognition, comparative neurobiology, approximate number system

## Abstract

The ability to represent, discriminate, and perform arithmetic operations on discrete quantities (numerosities) has been documented in a variety of species of different taxonomic groups, both vertebrates and invertebrates. We do not know, however, to what extent similarity in behavioral data corresponds to basic similarity in underlying neural mechanisms. Here, we review evidence for magnitude representation, both discrete (countable) and continuous, following the sensory input path from primary sensory systems to associative pallial territories in the vertebrate brains. We also speculate on possible underlying mechanisms in invertebrate brains and on the role played by modeling with artificial neural networks. This may provide a general overview on the nervous system involvement in approximating quantity in different animal species, and a general theoretical framework to future comparative studies on the neurobiology of number cognition.

## Introduction

The ability to represent, discriminate, and perform operations on discrete quantities has been documented in species of different taxonomic groups, proving that non-symbolic numerical cognition is not a human prerogative (Vallortigara, 2015, 2017; Butterworth et al., 2018). Since the early work by Koehler (1951), there has been accumulating evidence that both vertebrates and invertebrates are able to use non-verbal and non-symbolic quantities for relative numerosity judgments and to compute arithmetic operations. Among mammals, primates have been the main focus of research on comparative numerical cognition and not only different species demonstrated to possess this ability (Thomas and Chase, 1980; Brannon and Terrace, 1998; Anderson et al., 2005; Beran et al., 2008) but also they showed patterns of behavior very similar to those of humans (Cantlon and Brannon, 2006; Merten and Nieder, 2009). Numerical competences have been reported in rats (Davis and Albert, 1986), dogs (Ward and Smuts, 2007), cats (Pisa and Agrillo, 2009), lions (McComb et al., 1994), elephants (Perdue et al., 2012), and several other mammals. The main feature reported by these behavioral experiments is the animals' capacity to perceive the numerosity of sensory stimuli in an analog and noisy way, relying on an isomorphism between the physical quantity and its internal representation. Similar results have been obtained also in birds (e.g., Lyon, 2003; Templeton et al., 2005; Rugani et al., 2009, 2015; Scarf et al., 2011; Vallortigara, 2012; Kirschhock et al., 2021), reptiles (e.g., Gazzola et al., 2018; Miletto Petrazzini et al., 2018), amphibians (e.g., Uller et al., 2003; Stancher et al., 2015), and fish (e.g., Agrillo et al., 2010; Potrich et al., 2015, 2019), which constitute more phylogenetically distant taxa with respect to humans. Even more compelling is the evidence that comes from studies on invertebrates (for a complete review, see Skorupski et al., 2018; Bortot et al., 2020a) that unlighted further how widespread and biologically relevant is the ability to perform estimation and operations with numerical quantities in the animal kingdom. Besides, such evidence reveals how numerical cognition abilities can be implemented in small brains without the cortex (Giurfa, 2019).

Overall, these studies suggest the existence of an ancient mechanism which has been conserved through evolution and which is at the heart of the ability of animals to approximately estimate the numerosity of stimuli, which is the number of elements contained in a set (Brannon and Merritt, 2011). This is what has been dubbed Approximate Number System (ANS) and which is chiefly characterized by being based on Weber's law, the psychophysical rule which formalizes the systematic relationship that exists between the magnitude of physical stimuli and the power of discriminating them. According to Weber's law, the ratio of the increment threshold to the background intensity of a stimulus is a constant.

The animal numerical competence must undoubtedly be associated with increasing probability of survival and reproduction (Nieder, 2019, 2020a), thanks to the capacity to better discriminate and avoid predators or enemies (McComb et al., 1994), to navigate in the environment, and to find food, social partners, and sexual mates (Lyon, 2003). The ability to discriminate quantity in a natural environment seems to be so biologically crucial that even unicellular organisms are provided with mechanisms of “quorum sensing” to fulfill it (Waters and Bassler, 2005). An open question, however, is whether this numerical competence emerges out of a homologous trait conserved from the last common ancestor of all these species or if instead a process of convergent evolution has led to its development independently in each of these vertebrates and invertebrates (Ferrigno and Cantlon, 2017).

Aside from ANS, the existence of another mechanism possibly involved in the non-symbolic representation of numbers, namely, the Object Tracking System (OTS), has been hypothesized (Feigenson et al., 2004; Piazza, 2010). The OTS should account for simultaneous, fast, and unconscious visual perception of small sets of objects with an upper bound of three to four items, a process also known as “subitizing” (though whether subitizing and OTS are identical processes is unclear). It is well-differentiated from ANS in being precise and dependent on the absolute number of items, while in contrast numerical ratio is the main feature of ANS. More importantly, OTSs are not dedicated to numerosities, but they are implicitly represented in the objects tracked. The discussion on whether there exist two systems is still open (Nieder, 2019; and see for animal research, e.g., Rugani et al., 2013a), a possibility being that the peculiar phenomena of subitizing can be explained by ANS without any need to postulate the existence of a second mechanism, for scalar variability for small numbers would be so low that it may appear as corresponding to precise counting (Gallistel and Gelman, 1992; Dehaene and Changeux, 1993; Vetter et al., 2008). However, in this review we shall focus only on ANS and its neural representations, since we are interested in reviewing this more general system, which is primarily characterized by the perceptual feature of the ratio effect, and several evidences support it as the only truly quantitative system.

The ANS is the biological mechanism underlying the intuitive and noisy estimation of quantity that Dehaene has called—actually taking the expression from Tobias Dantzig—“the number sense” (Dehaene, 2011). This epithet precisely lingers on the sensory, thus approximate and spontaneous, component of this ancient numerical competence. It involves the estimation of both small and large sets of quantities, without any upper boundary (Cordes et al., 2001). It has to be noticed that while vertebrates with bigger brains succeed in managing larger numerosities without constraints, it has long been maintained that invertebrates would tend to have a limit of four objects that they are able to discriminate and onto which they can be made arithmetic (Skorupski et al., 2018); however, recent studies seem to extend abilities at least up to six elements (e.g., Howard et al., 2018; see for a review Bortot et al., 2020a). Approximate number system is a presymbolic and preverbal system; indeed, in humans it seems to fulfill the peculiar role of preceding ontogenetically and giving foundation to the symbolic representation of numbers (Gallistel and Gelman, 1992, 2000; and work with innumerate indigenous peoples would support this, e.g., Gordon, 2004; Pica et al., 2004). It explains why 5-year-old children without any scholastic education are able to compare large sets of stimuli both visually and acoustically, whether simultaneous or sequential, excluding any influence by spontaneously learned symbolic knowledge (Barth et al., 2005). Additionally, ANS is employed both in simultaneous and sequential numerical tasks and across sensory modalities (Hauser et al., 2003; Jordan et al., 2005, 2008). Indeed, numerosity can be considered an amodal property of a physical set (Giaquinto, 2018), and as such, it can be displayed through the physical sense more relevant for a given moment and a particular species.

The main signature of the ANS is the ratio effect, i.e., the fact that the ability to differentiate two quantities—in terms of accuracy and rapidity—is a function of their ratio, rather than their absolute value. This effect is accounted for by Weber's law and emphasizes that a perceptual process is involved in numerical judgments (Moyer and Landauer, 1967). The discrimination of numerosity obeys Weber's law in humans (Moyer and Landauer, 1967) as well as in other vertebrate (Mechner, 1958; Ditz and Nieder, 2016) and invertebrate (Skorupski et al., 2018) species. The dependence on the ratio between numerosities has two sequels called distance effect and size effect. The distance effect consists in the greater discriminability of two distant numbers rather than two close ones. The size effect instead refers to the reduced accuracy in differentiating at a given numerical distance two large numbers with respect to two smaller ones (Dehaene and Changeux, 1993).

Several behavioral evidences report a non-linear dependence between the perceptual and numerical objects. Two hypotheses have been put forward as an explanation for these phenomena: the scalar variability proposal and the logarithmic proposal (Brannon and Merritt, 2011). The first is mainly endorsed by Gallistel and Gelman (1992); according to them, numerosity is characterized by a linear scale and variance is proportional to numerosity itself. This implies that higher numerosities have noisier representations (Merten and Nieder, 2009). The second hypothesis states a constant variability, but according to it mental magnitudes are proportional to the logarithm of the objective magnitude (Dehaene, 2003). Despite both hypotheses being congruent with the behavioral data, their disentangling could be made looking at the neural encoding of numbers, which according to a principle of parsimony seems to be better described by a logarithmic scale (Dehaene and Changeux, 1993; Nieder and Miller, 2003; Ditz and Nieder, 2016).

Another important aspect of numerical cognition is that the preverbal discrimination of numerosity has been hypothesized to belong to a general magnitude system, responsible for the representation of space, time, and quantity (Gallistel, 1989; Walsh, 2003). Indeed, several studies demonstrated that the temporal and numerical features of stimuli can interact and bring to indistinguishable psychophysical functions both following Weber's law, suggesting a common mechanism for counting and timing (Meck and Church, 1983; De Corte et al., 2017). Discrete quantities could be represented as continuous magnitudes (Gallistel and Gelman, 2000), which are the same currency used to quantify duration.

Concerning this tight relationship of numerosity with other systems of magnitude, some authors have advanced the hypothesis that continuous magnitudes which naturally covary with numbers—such as area, perimeter, and density—might be the leading information processed in tasks on numerosity (Leibovich et al., 2017). Several studies try to control for these variables, at least once at a time so as to tease apart their impact on numerosity tasks from effects of ANS (Rugani et al., 2011, 2013b; DeWind et al., 2015; Testolin et al., 2020a). Animals for example should be aware of the direction of the correlation between numerosity and the alternative continuous variable, in order to rely on the second one to complete those tasks (Ferrigno and Cantlon, 2017). However, one recent experiment in bees demonstrated how problematic it is to assess whether an animal is actually learning numerical information instead of the continuous one (MaBouDi et al., 2021), but mostly that behaviors relying on continuous cues could reproduce signatures indicative of numerical cognition. Indeed, bees that were trained to discriminate numerosity according to conventional paradigms on further controls revealed to rely on continuous quantities. We cannot exclude a narrow association of numerosity and these covarying aspects of physical stimulation, and indeed the difficulty in distinguishing them is also proved by the fact that these variables are similarly represented (Meck and Church, 1983); however, this is not sufficient to exclude the existence of a numerical competence among the others. Indeed, many (training) studies went at great length to exclude non-numerical factors and to demonstrate real numerical quantity discrimination (see for a review Nieder, 2019).

Given all these theoretical and behavioral premises, in this review we shall focus on the neural underpinnings of the number sense. While much has been reported and discussed about the behavioral proofs of this numerical capacity, the literature around the neural correlates is still in its infancy and presents discrepancies, which are testified also by the slow growth with respect to the early studies regarding this topic (Thompson et al., 1970). In particular, little attention has been devoted to the comparative aspects of number representation in the brains. On one side, we have behavioral evidence for number representation spanning from primates to cephalopods and insects, and striking suggestions that the basic signatures of the ANS system would be apparent for all these organisms. On the other side, we know that the nervous systems of all these creatures are enormously different, and most of the direct neurobiological evidence comes from a bunch of organisms, mostly non-human primates and one species of birds.

## Numerosities and Other Kind of Magnitudes in the Brains

From a neurobiological perspective, most studies on number cognition focused on primates' cortical areas and on the nidopallium caudolaterale (NCL) of corvids, which is supposed to be equivalent to the primates' prefrontal cortex (Mogensen and Divac, 1982; Divac et al., 1985; Güntürkün, 2005; Nieder, 2018; Stacho et al., 2020). In comparison, few studies investigated the contribution of subcortical (sub-pallial) regions. This is partly due to the idea that number cognition should be regarded as an advanced cognitive skill, implying involvement of associative and “higher-order” neural mechanisms located in the pallial territory of vertebrate animals. However, as noted in the previous section there is substantial evidence for the “number sense” (ANS) being deeply rooted into primary sensory mechanisms and highly shared among phylogenetically distant animal species, from humans to honeybees (Butterworth et al., 2018; Bortot et al., 2020a). Moreover, several authors argued for the existence of a general magnitude system, of which discrete (countable) numerosity would be only a part, dealing with quantity information whatever the format (continuous or discrete) and the domain (space, time, and number; Gallistel, 1989; Gallistel and Gelman, 2000; Walsh, 2003; Lourenco and Longo, 2010; Merritt et al., 2010; Rugani et al., 2015, 2020; Bortot et al., 2020b). Numerosity perception mirrors to a certain degree sensory activity, appearing to obey to Weber's law and showing the same adaptation phenomena that we know to exist for perceptual features such as color or stimulus orientation (Burr et al., 2018; see also Burr and Ross, 2008). Furthermore, species that do not possess pallial homologs, such as insects, or even artificial neural networks seem capable of quantity discrimination on the basis of (discrete) numerical information (Skorupski et al., 2018).

**Figure 1:**
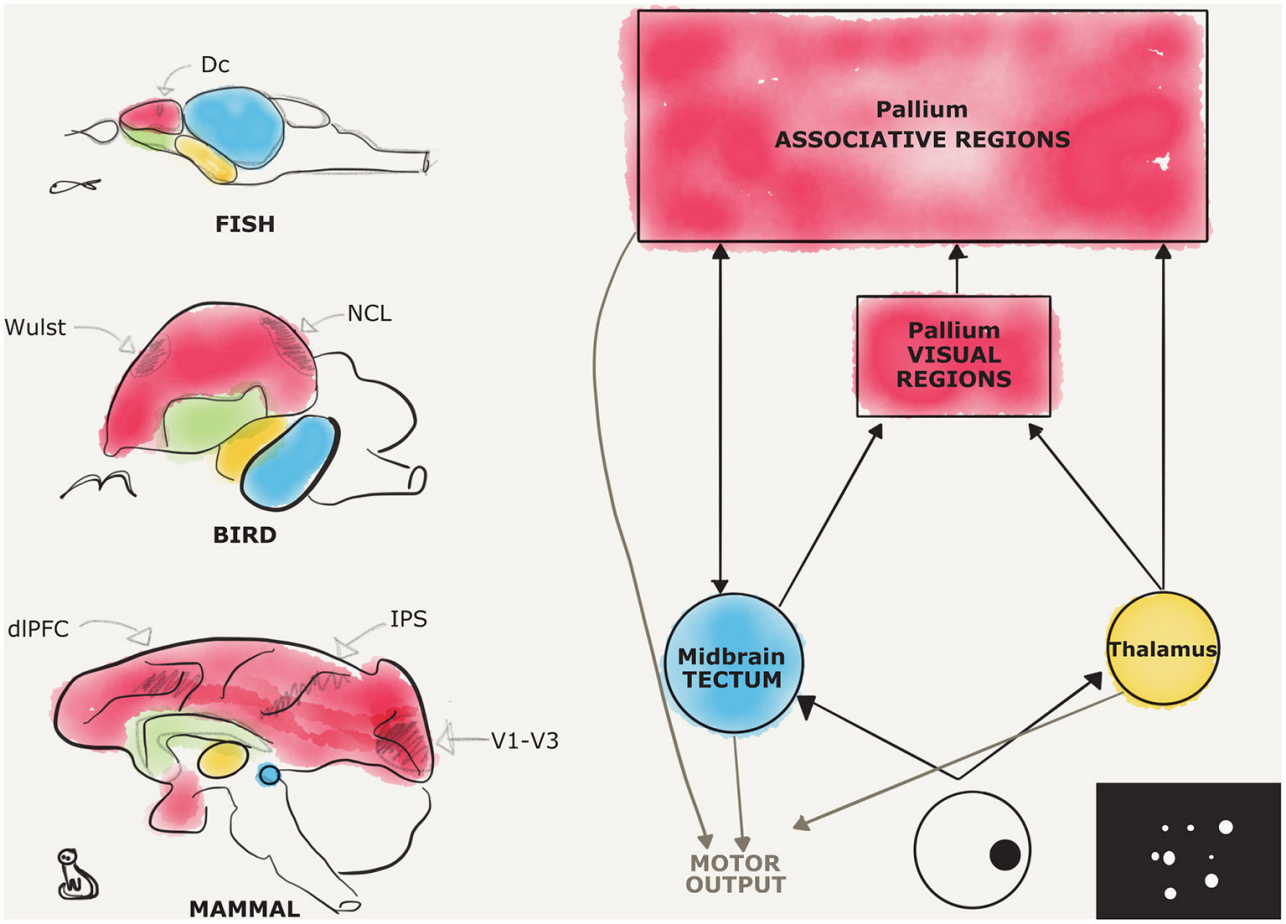
Candidate regions for number processing in the vertebrate brains, a comparison. Schematic representations of different vertebrate brains are provided on the left, respectively, from the top, for fish, birds, and mammals. Pallial territories are depicted in red, subpallial in green, thalamic in yellow, and midbrain in blue. Fine subdivisions of the pallium are depicted in pencil, the dorsocaudal division (Dc) in fishes, the visual Wulst, and the nidopallium caudolaterale (NCL) in birds, the dorsolateral prefrontal cortex (dlPFC), the intraparietal sulcus (IPS), and the striate and extrastriate visual cortices (V1–V3) in mammals. On the right, a diagram depicts in a simplified fashion the two main visual pathways, and an example of a stimulus used for studying number estimation is shown. The interconnections between the different hubs are depicted with black arrows, while motor afferences are shown in gray.

Here, we will review evidence for magnitude representation (both discrete and countable, and continuous) following the sensory input path from primary sensory systems to associative pallial territories (see Figure 1). This may help give a general overview on the central nervous system involvement in numerical cognition in different animal species and provide a general theoretical framework to future comparative studies on the neurobiology of number cognition.

### Primary Sensory Systems

Rising evidence points to a primary sensory involvement in estimating magnitudes. As noted above, numerosity perception is susceptible to sensory adaptation similarly to primary sensory properties such as size or color (Burr et al., 2018; for a review see Burr and Ross, 2008). Numerosity estimates increase after a period of adaptation to small sets, and the opposite occurs for large sets. Therefore, as it is the case for other primary sensory properties, sensory systems should be able by themselves of coarse number estimates (Burr and Ross, 2008).

Number and size representations are likely to be associated in the brain. Using high-field functional magnetic resonance imaging (fMRI), Harvey et al. (2013, 2015) showed that in the human posterior parietal lobe there are regions responsive to different stimulus sizes and that both sizes and numerosities are topographically organized into maps. As it is the case for primary sensory and motor cortices, preferences in the neural response for similar numerosities, as well as for similar sizes although in distinct subpopulations, are contiguous and gradually change across the cortical territories.

#### Tectofugal Pathway

The tectum is a midbrain structure shared among vertebrates, which is involved in sensorimotor functions (Ingle and Sprague, 1975). A crucial relay of visual and auditory systems, the tectum receives direct sensory input and is reciprocally connected with the forebrain and the spinal cord (see Figure 1).

The optic part of tectum, also known as superior colliculus in mammals, is involved in competitive visual stimulus selection. It controls rapid orienting behaviors, directly selecting and estimating the value of the target stimulus (Gardner and Lisberger, 2002; McPeek and Keller, 2004; Lovejoy and Krauzlis, 2010; Mysore et al., 2011; for a comparative perspective see Mysore and Knudsen, 2011). The tectum represents the visual space and converts a visuotopic sensory map into a map of directed motor outputs (Nevin et al., 2010). It appears to be organized into layers, with the superficial layers receiving direct information from retinal ganglion cells (Sajovic and Levinthal, 1982; Del Bene et al., 2010). Some neurons in this region tune to distinct visual properties such as looming, moving, or size. Actually, size selectivity is an emergent property of the intratectal circuitry (Sajovic and Levinthal, 1982; Del Bene et al., 2010). Seminal work on anurans' prey-catching behavior revealed tectal selectivity for many different visual features. In particular, some neurons appear to be selectively sensitive to different object sizes (Ewert and Gebauer, 1973; Cervantes-Pérez et al., 1985; Ewert, 1987).

Neurobiological studies on zebrafish larvae (*Danio rerio*) revealed that the optic tectum encodes and classifies object size. Different subpopulations of tectal neurons are tuned to small or large objects, and their receptive fields are shaped through direct afferent input from retinal ganglion cells (Preuss et al., 2014; Barker and Baier, 2015).

Estimations of immediate-early gene (IEG) expression in adult zebrafishes confirmed an optic tectum involvement during stimulus size changes: using a habituation/dishabituation paradigm, it was found that changes in stimulus size during the dishabituation phase were inversely proportional to *c-fos* expression in the tectum (Messina et al., 2020a).

In pigeons (*Columba livia*), electrophysiological recordings in the optic tectum revealed the presence of some neurons that modulate their activity in response to stimulus size changes (Gusel'nikov et al., 1971).

Receiving direct visual information from the retina, the optic tectum progressively, from the outer to the deeper layers, segregates information and performs a fast stimulus classification based on sensory parameters (Luksch, 2003; Wylie et al., 2009). Subsequently, it can promote itself a behavioral response, while the classified visual information proceeds to higher-order visual and associative areas.

Quick estimation of stimulus magnitude, such as number discrimination, is often essential to performing adaptive behavior in the shortest time. For instance, group-living animals, such as some fish or anuran tadpole, in the presence of a predator need to rapidly estimate the largest group of conspecifics and to aggregate with it, thus maximizing their survival rate (Hoare et al., 2004; Agrillo et al., 2008; Gómez-Laplaza and Gerlai, 2011; Balestrieri et al., 2019). The tectum could be an ideal candidate region to perform fast and coarse number discrimination classifying sensory information, in this case larger and smaller number of conspecifics, and give rise directly to motor output toward the larger group.

Evidence from another sensory domain has revealed the presence of neurons in the torus semicircularis that appear to count auditory signals, dubbed interval-counting neurons (Rose, 2018). Male toads, which compete with each other to mate, usually emit complex advertisement calls comprised of several different pulses to attract females. Females evaluate the complexity of the calls in order to choose the partner. In the anurans inferior colliculus, the main tectal nucleus of the auditory pathway homolog among vertebrates, whole-cell recordings revealed that a first pulse inhibits the interval-counting neurons, while as progressively other pulses arrive the cells depolarize and spike when a certain number of pulses (threshold) is reached (Rose, 2018). Note, however, that the neurons in the anuran torus semicircularis require very specific inter-pulse intervals and do not generalize across variations of interval times. Therefore, it is uncertain whether such neurons can be regarded as true “counting neurons.”

Indirect evidence suggests a subcortical involvement in quantity estimates even in humans (Collins et al., 2017). Exploiting the separation of the visual signals coming from each eye until layer IV of the primary visual cortex (V1), pairs of stimuli were presented sequentially either to the same or to different eyes. Human participants were asked to compare the numerical magnitude within each pair (same/different). Numerosity estimation was facilitated in the monocular condition for large ratios (3:1 or 4:1) for both small (<4) and large (>5) numerosities. Given the prestriate separation of monocular signals, ratio-dependent monocular judgments could have benefited from a substantial subcortical contribution, for beyond layer IV of V1 very few monocular neurons exist (Horton et al., 1990; Menon et al., 1997; Bi et al., 2011).

Such findings fit in well with studies on number discrimination in newborns (Izard et al., 2009). Forty-eight-hour-old newborns are able of number discrimination when the ratio is 3:1 or larger. The collothalamic pathway, which comprises the tectum, is functional already at birth, while the geniculocortical pathway probably becomes fully functional later in development, around 2 months of age (Bronson, 1982; Atkinson, 1983; Braddick et al., 1986; Atkinson and Braddick, 1989; Atkinson et al., 1992). This may suggest a major involvement of the collothalamic pathway in newborn number cognition.

One electroencephalogram (EEG) study in young children (3–10 years) revealed little cortical involvement for numerosity processing, which gradually increases throughout development (Park, 2018). From this perspective, coarse numerical abilities could be implemented in subcortical (possibly inborn see, e.g., Di Giorgio et al., 2019) mechanisms. As in the case of face perception (Johnson, 2005; Di Giorgio et al., 2017; Lorenzi and Vallortigara, 2021; Rosa-Salva et al., in press), an inborn subcortical mechanism would provide the basics for number cognition, while subsequent experience and learning would then capitalize on this mechanism to shape the more sophisticated cortical computations for complex numerical tasks. Note, however, that Izard et al. (2008) reported a cortical involvement of number representation in 3-month-old infants.

Lesion studies in primates showed that even in the absence of the striate cortex (V1) the input from the superior colliculus still reaches the cortical dorsal stream (Rodman et al., 1989; Rosa et al., 2000). Such direct connection may support a superior colliculus involvement in numerical abilities early in development. Functional magnetic resonance studies also confirm a midbrain activation during numerical tasks in human adults, which progressively enhances with the increase in the number of items (Piazza et al., 2002).

Altogether, this evidence strongly supports a midbrain involvement in coarse number discrimination among vertebrates. From this perspective, the tectum appears to be an ideal candidate region for fast quantity discrimination, highly conserved through phylogeny, and early maturing in ontogeny.

#### Thalamofugal Pathway

Another important hub and relay for primary sensory information is in the dorsal diencephalon, the thalamus. Different thalamic nuclei receive primary sensory information from different sensory modalities and send it to multiple cortical fields (Herrero et al., 2002). Other thalamic nuclei, instead, act as motor relays connecting basal ganglia to motor cortices (Evarts and Thach, 1969; Kurata, 2005).

The lateral geniculate nucleus (LGN), a visual thalamic nucleus part of the geniculocortical visual system, is a layered structure that, similarly to the optic tectum, receives direct input from the retinal ganglion cells (Perry et al., 1984). The LGN is highly conserved among vertebrates, and its superficial layers are topographically organized (Glees, 1941; Crossland and Uchwat, 1979). It projects to the primary visual areas in the pallium (Rezak and Benevento, 1979; see Figure 1).

Responses of neurons in the LGN of cats (*Felis catus*) to different light intensities were shown to obey Weber's law (Podvigin and Chueva, 1977; Podvigin and Elefandt, 1983). Weber's law being a signature of the ANS, this could suggest an involvement of the LGN in coarse number (quantity) estimates. Strengthening such hypothesis, IEG's expression in zebrafish revealed a thalamic involvement in number processing and quantity estimation (Messina et al., 2020a). After a habituation phase with either a small (3) or a large (9) number of dots, fish faced a change in numerosity during a dishabituation phase. Then, *c-fos* and *egr-1* expression following the dishabituation were quantified in the brain. Intriguingly, thalamus expression of both genes was significantly affected by different directions of change: after a change from large to small numbers of dots, the IEGs' expression decreased in the thalamus, while it increased after a change from small to large number of dots (Messina et al., 2020a).

The thalamus sends visual and auditory sensory inputs to the visual and auditory regions in the pallium in all vertebrate brains (Medina and Reiner, 2000; Bloch et al., 2020). The LGN in mammal relays retinotopic information to the primary visual cortex (V1; Sefton et al., 2015), and similarly thalamic nuclei in the other vertebrates topographically project visual information to a portion of the dorsal pallium (Karten, 2015; Suryanarayana et al., 2017, 2020). Different neurons in these pallial visual regions are sensitive to different visual properties such as orientation or color.

Interestingly, change in numerosity of visual stimuli in zebrafish also revealed selective expression of IEG in a dorsocentral division of the pallium (Messina et al., 2020b). Such selectivity could be the result of the thalamic as well as tectal input to pallial regions (Bloch et al., 2020). Further studies are needed to clarify the precise pattern of afference to the dorsocentral division of pallium.

Evidence in human adults also suggests a role of early pallial/cortical regions of the thalamofugal pathway in visual magnitude estimations. Primary visual cortices, both striate and extrastriate, are selectively involved in response to magnitudes of visual items. Positron emission tomography (PET) as well as fMRI studies showed an involvement of middle occipital extrastriate visual cortices when subjects were explicitly required to enumerate dots in a visual array (Piazza et al., 2002; Demeyere et al., 2014). However, numerosity-sensitive signatures arise from early visual cortices also when subjects were only passively exposed to different numbers of dots. A medial occipital cortical component in event-related potentials was observed, monotonically modulated by number 75–90 ms after stimulus presentation (Park et al., 2016). This early response likely reflects an involvement of primary visual cortices (V1, V2, and V3) when subjects were simply exposed to numerosity, as confirmed by a study in which different numerosity arrays were presented in the upper or in the lower visual hemifield, taking advantage of the peculiar organization/polarization of the primary visual cortices around the calcarine sulcus (Fornaciai et al., 2017). Visual cortices distribute around the sulcus so that the upper bank receives retinotopic input from the lower visual hemifield, while the opposite happens for the lower bank. This distribution is apparent from the polarity inversion of event-related potentials arising from these regions by EEG (Ales et al., 2013; Kelly et al., 2013a,b). An early robust inversion of polarity was detected for numerosities presented in either the upper or the lower visual hemifield, suggesting the origin of the response in V2 and/or V3. Importantly, these early responses were more selective to numerosity than to other continuous variables that covary with numerosity, such as overall area or contour length (Park et al., 2016; Fornaciai et al., 2017).

Remarkably, other studies showed that the activity pattern measured in the visual striate cortices was different when the task required to estimate the number or the overall surface of dot arrays (Fink et al., 2001; Castaldi et al., 2019). This suggests that different mechanisms may be involved in estimating continuous and discrete quantities already at the level of the striate cortex. Employing a set of stimuli rigidly controlled for continuous physical variables, the occipital involvement in encoding discrete variables (number) was not influenced from continuous variables (DeWind et al., 2019).

Evidence from the auditory domain strengthens the hypothesis of number as a primary perceptual feature, for similarly to the visual cortex, the auditory cortex was found to be selectively involved when estimating the number of sequential sounds presented (Cavdaroglu et al., 2015). From this perspective, sensory pathways could play a prominent role in coarse number estimations and, subsequently, the associative parietal cortices could further process numerosity categorized along the sensory pathways. Indeed, evidence from the visual domain suggests a pathway following the occipitoparietal stream for numerical information processing (Roggeman et al., 2011).

### Associative Regions

A considerable amount of evidence points toward number being an abstract concept emerging from associative pallial regions. A pallial involvement in numerical cognition has been argued for in both primates and corvids. Although conceptually this perspective seems to exclude the idea of number as a primary sensory feature early encoded in the sensory pathways, from a neurobiological perspective the two ideas may not be mutually exclusive. It could be that while numerousness is extracted early in the sensory stream it is further processed and manipulated in more associative areas when the numerical task is more demanding. Here, we will briefly review evidence for associative pallial regions' involvement in coarse number estimation.

#### Posterior Parietal Cortex

The posterior parietal cortex in mammals is an integration hub where visual and auditory information is combined to the somatosensory one (see Figure 1). It comprises the dorsal visual stream and the cortex around the intraparietal sulcus (IPS). The dorsal visual stream receives retinotopic information from striate and extrastriate cortices (V1–V3; Mishkin and Ungerleider, 1982). The IPS is particularly involved in merging sensory information to plan movements of the eyes or the limbs (Cohen and Andersen, 2002). Moreover, important outputs are sent from the posterior parietal lobe to motor and premotor areas of the frontal lobe.

Functional MRI studies on human adults revealed IPS activity in numerical estimation, using both visual and auditory stimuli (Castelli et al., 2006; Piazza et al., 2006). Crucially, such activity does not depend on explicit numerical task demands. Simple exposure to numerosity elicits activation in the IPS (Piazza et al., 2004). Intraparietal sulcus involvement has been detected also in 4-year-old children and earlier in 3-month-old infants measuring event-related potentials (Temple and Posner, 1998; Cantlon et al., 2006; Izard et al., 2008; Kersey and Cantlon, 2017). A similar pattern of activation, although more generally distributed in the parietotemporal cortex, was observed when dogs (*Canis familiaris*) passively watched set of dots in an fMRI study (Aulet et al., 2019). Activity measured in this region crucially depended on the ratio between different numerosities, obeying Weber's law.

In human and non-human primates, two subregions of the IPS have been reported to be involved in number cognition, the lateral intraparietal area (LIP) and more deeply inside the sulcus the ventral intraparietal area (VIP; for a review see Nieder and Dehaene, 2009).

One study of single-cell recordings in macaques' (*Macaca mulatta*) LIP revealed the presence of neurons that seem to act as an abacus (Roitman et al., 2007). These neurons increase their firing rate progressively as the number of items increases. It is worth stressing, however, that this was reported for a task in which numerosity had not to be discriminated by the monkeys. Nonetheless, as we will see more thoroughly in section Neural Networks, numerical estimation has been proposed to be computationally based on two possible neuronal mechanisms, either a summation one or a number-selective one (Chen and Verguts, 2013).

The summation mechanism postulates the existence of neurons that would respond monotonically to numbers, increasing their firing rate with increasing numerosity (Verguts and Fias, 2004). The response of the neurons recorded in the macaque's LIP acts in a summation fashion. Similarly do the interval-counting neurons observed in the anuran inferior colliculus (Rose, 2018). These pieces of evidence point toward a possible summation coding mechanism extracting numerosity from the environment in different neural substrates at different processing levels.

On the other hand, the number-selective mechanism postulates the existence of number neurons that respond to a preferred numerosity and to other numerosities proportionally to the distance from the preferred one (Verguts and Fias, 2004). Single-cell recordings in monkeys' VIP revealed neurons tuned to a specific numerosity, dubbed number neurons (Nieder et al., 2002; Sawamura et al., 2002; Nieder and Miller, 2004; Nieder and Merten, 2007). Such tuning seems to be not affected by continuous variables, being instead specifically elicited from the discrete quantity represented. The selectivity in the neuronal response reaches its peak for the preferred numerosity, while adjacent numerosities elicit weaker responses coherently with the distance from the preferred numerosity. Such property exhibited by the neuronal response would explain the behavioral distance and size effects, thus obeying Weber's law (Nieder, 2020b). These number neurons respond also when the preferred numerosity is passively perceived and in number-naïve monkeys that did not experience any number training (Viswanathan and Nieder, 2015). This evidence would be in agreement with the hypothesis of an unlearned number-selective mechanism underlying number estimation.

The two mechanisms, summation and number-selective, are not however mutually exclusive. For example, a three-stage computational model has been proposed for number estimation (Dehaene and Changeux, 1993; Verguts and Fias, 2004) that would account for a role of both mechanisms. The first stage of the model would involve an item-location map, where discrete items are encoded with respect to the different spatial location in which they appear. Secondly, the location information passes through a summation mechanism, in which the different discrete locations are accumulated and transformed into a quantity. Finally, the quantitative information is generalized and transformed into an abstract number-selective code. An fMRI study exploiting an adaptation paradigm found plausible neural candidates for these three different stages, along an occipito-parietal gradient (Roggeman et al., 2011). From inferior- through middle-occipital gyrus to the superior parietal lobe, BOLD responses modulated coherently with the location map, the summation code, and finally the number-selective code (Roggeman et al., 2011).

However, evidence from lesion studies suggests some caution in interpreting results from number-selective neurons (DeWind et al., 2019). When LIP or VIP is selectively inactivated in monkeys, a general impairment is observed in numerical as well as in color discrimination tasks. No specific effect on number over color discrimination was detected. This could be due to the role played by the IPS in general attentive or decision-making processes. Evidence that these neurons are behaviorally relevant was previously provided in another lesion study by Sawamura et al. (2010). Further investigations therefore need to disentangle the exact IPS involvement in numerical estimation tasks.

#### Mammalian Prefrontal Cortex, Avian Endbrain, and Fish Dorsocentral Pallium

The prefrontal cortex (PFC) in mammals is widely recognized as a fundamental hub for high-level and flexible cognitive control, so-called executive functions. Executive functions comprise skills ranging from decision-making, inhibitory control over behavior, to planning (Fuster, 2015). Strategically, the PFC receives sensory information from all the different modalities and projects back to them (Pandya and Yeterian, 1991). It is a crucial site where sensory input and motor output are combined (see Figure 1).

In human and non-human primates, different evidences suggest the existence of a frontoparietal network involved in numerical estimations (Nieder et al., 2002). A portion of the lateral PFC and the IPS are intensely interconnected with each other (Selemon and Goldman-Rakic, 1988). Similarly to the IPS, populations of neurons in the dorsolateral PFC selectively respond to preferred numerical proportions (Jacob and Nieder, 2009). Using an fMRI adaptation paradigm with human adults, PFC and IPS both showed a similar response to changes in the numerical ratio. Such response was proportional to the change from the preferred ratio (Jacob and Nieder, 2009).

Single-cell recordings in monkeys confirmed that number-selective neurons in the PFC follow Weber's law, as it is the case for IPS' number neurons (Nieder and Merten, 2007). Differently from parietal neurons, however, frontal number neurons seem to be directly related to the behavioral performance in the numerical task, for a correspondence has been observed between incorrect trials and a decrease in the spiking rate for the preferred numerosity (Nieder and Merten, 2007; Viswanathan and Nieder, 2015).

Interestingly, PFC number-selective neurons respond to their preferred numerosity both when presented visually or auditorily (Nieder, 2012). While in the IPS different neurons respond to the same numerosity presented in the two modalities, a large proportion of neurons in the PFC responded to both modalities suggesting a supramodal generalization step achieved in this region. Moreover, strengthening the role of the abstract generalizator played by the PFC, a direct comparison between neuronal responses in the PFC and IPS revealed that frontal neurons are less influenced from continuous variables that covary with discrete numerosities (Nieder and Miller, 2004). Moreover, the difference in the latency of response between the earlier parietal (100 ms) number neurons and the later frontal ones (160 ms) strongly suggests a hierarchy between these two regions for number estimations. It has been hypothesized that IPS is involved in extracting number information from the sensory input, while PFC further processes and generalizes such information from a behavioral goal-directed perspective (Nieder, 2016).

Comparative studies revealed extraordinary functional and organizational similarities between the PFC and a caudal part of the pallium in birds, the caudolateral nidopallium (NCL; Mogensen and Divac, 1982; for comprehensive reviews see Güntürkün, 2005; although Stacho et al., 2020, showed that the NCL is explicitly not layered and thus similarity with mammalian PFC is likely to reflect homoplasy rather than homology for this region; see Figure 1). Similarly to primates' PFC, responses to numbers were measured in single cells in the NCL of corvids (Ditz and Nieder, 2015). Number-selective neurons were found to increase their firing rate to a preferred numerosity while obeying Weber's law (Ditz et al., 2018). Moreover, such number neurons were found also in number-naïve birds, confirming their unlearned tuning properties across mammals and birds (Wagener et al., 2018).

A pallial involvement in numerical changes was found also in the telencephalon of zebrafishes (Messina et al., 2020a). A dorsocentral division of zebrafish caudal telencephalon was found to be selectively involved during numerosity changes (Messina et al., 2020b; see Figure 1). This pallial region, as we speculated in section Thalamofugal Pathway, could be a visual area. It could also be a more associative nidopallial-like region involved in more flexible cognitive skills and behavioral control. However, further studies need to elucidate the peculiar afferent and efferent connections' pattern of such telencephalic division of zebrafish brain.

Overall, it can be reasonably stated that a general involvement of a pallial network has been revealed across different classes of vertebrates in number estimation (Figure 1).

## Invertebrates

As already noted, the evidence coming from invertebrates constitutes a strong argument in favor of a widespread numerical competence, deconstructing the priority that the mammalian cortex has held even in the field of comparative studies on numerosity. Since small brains in the order of one million neurons can discriminate numerosity among other continuous variables (Gross et al., 2009; Bortot et al., 2019), it seems plausible that small circuits and somewhat restrained computational resources are sufficient in order to accomplish it. Furthermore, looking for homologs of the prefrontal or parietal cortex in other species might not be necessary in order to study the neural correlates of this ability. It may represent a possibility, however, that deep homologies (see Held, 2017) exist as to the mechanisms underlying encoding of discrete and continuous quantity among widely separate taxa.

While literature presents several data about the behaviors of invertebrates in numerical tasks (review in Bortot et al., 2020a), almost nothing is known about their neural implementations. However, we can try to focus on the strategies employed in these tasks in order to draw hypotheses about their underlying biological mechanisms.

Bees (*Apis mellifera*)—which have been used as the main animal model for studying numerical competence in invertebrates—seem to make use of a sequential visual scanning (Skorupski et al., 2018; MaBouDi et al., 2020), which means that they process stimuli in a sequential order through active visuomotor exploration. This has been supported by their failure in simple tasks when stimulus presentation is limited in time (Nityananda et al., 2014), the increase in time needed for discriminations for higher numerosity patterns (MaBouDi et al., 2020), the documented scanning behaviors from a distance of only few centimeters from stimuli when the animals have to avoid specific numerosities (Guiraud et al., 2018), and the fact that they exhibit serial processing of visual scenes even for other features of stimuli (Spaethe et al., 2006). Nevertheless, a possibility is that some combination of parallel and serial processing is involved in numerical tasks (MaBouDi et al., 2020). Besides, it cannot be excluded that also some vertebrates use similar strategies for numerical estimation (Dawkins and Woodington, 2000).

The use of sequential scanning appears to be in agreement with insects' photoreceptor architecture and the active vision shown by bees (Skorupski et al., 2018). Indeed, bees have a small visual acuity (Spaethe and Chittka, 2003; Rigosi et al., 2017) and possess 3,000–4,000 ommatidia (Spaethe and Chittka, 2003), which limit the visual scene perceived and thus the possibilities of parallel visual processing. Conversely, the fastest temporal response of their photoreceptors appears to be highly functional to this type of behavior (Skorupski and Chittka, 2010), thus profiting of a great amount of information collected through fast movements. In other words, sequential scanning might exploit the biological repertoire with a computational advantage.

An implication of this scanning behavior is the involvement of working memory circuits which must help the animal in tasks where counting is required to discriminate numerosities. Indeed, during sequential inspections of visual stimuli bees were very rarely observed to come back to rescan some items (MaBouDi et al., 2020). The one-to-one inspection of stimuli then requires a storing and progressive integration of information in working memory. Moreover, the decay span of working memory in sequential visual scanning can be the reason for the limited amount of items that bees seem to be able to distinguish (Skorupski et al., 2018).

Which areas of insects' brains might thus be more involved in numerical tasks? The visual system of vertebrates and insects shows similar morphogenetic mechanisms (Joly et al., 2016), especially for what regards the first somatotopic stages of visual processing accomplished by the retina, the optic tectum, and the LGN in vertebrates and by the retina, the lamina, the medulla, and the lobula in insects. Hypotheses about a phylogenetic conservation of these visual circuits have been advanced (Sanes and Zipursky, 2010).

Studies on the visual system of bees reported evidence for segregated pathways for processing of color and motion, respectively, in the distal lobula (layers 1–4) and in the proximal lobula (layers 5–6) (Paulk et al., 2008). Moreover, this functional segregation appears to be maintained along the anterior–posterior axis of the protocerebrum (Paulk et al., 2009), suggesting a differential computation of color and pattern information from achromatic motion and orientation features which are relevant for the more posterior motor areas.

Accordingly, numerical quantity could be encoded already in these early stages of visual pathway as a result of integration of sensory information from the achromatic pathway and recurrent circuits present at the level of the lobula (Haag and Borst, 2001) and of the medulla (Douglass and Strausfeld, 2003). Indeed, as noted above numerosity is mostly conceived as a primary sensory attribute (Burr and Ross, 2008), and recurrent circuits are known to constitute the ground for working memory (Wang, 2001). Alternatively, numerosity might be represented by the central complex (Giurfa, 2019), a higher cognitive area of insects' brain. The central complex is a center of sensory integration—mostly visual—but of importance also for motor control (Pfeiffer and Homberg, 2014). Furthermore, its involvement in short visual memory and in navigation strategies such as path integration (Stone et al., 2017) suggests a relevance also for numerical tasks.

However, a critical view must be taken in mind regarding the feasibility of a neural module dedicated to numerosity detection. The existence of neurons only devoted to the perception of a specific visual feature, such as numerosity, has been argued to be both not necessary and not easily experimentally detectable (Skorupski et al., 2018). The number of cells required to accomplish a similar task may be too exiguous to be precisely detected anatomically, and even the evidence for segregated feature processing in the optic lobes of insects does not exclude that these neurons might have more complex response patterns, thus responding to more than one attribute depending on the context. This argument points against too much enthusiasm for number-detecting neurons and the neuron doctrine, i.e., the tendency to ascribe to specific neurons the discrimination of particular physical properties (Barlow, 1972; von der Malsburg, 1994).

## Neural Networks

On the wake of this minimalist approach to numerical cognition, several theoretical models have tried on the one hand to experimentally validate if very low-level computations can accomplish tasks of numerical discrimination, on the other to describe plausible implementations of the numerical ability at the level of neuronal circuits.

Computational modeling and neural networks in particular are mathematical tools that enable to test and investigate biological and cognitive questions through simulations and exploiting artificial neural architectures that attempt to emulate the brain. Specifically, some of these models—namely, artificial neural networks—are architecturally inspired by the brain (Pillow and Sahani, 2019) and are used in a variety of contexts, such as for classification of images or decision-making and learning. Networks consist of thousands of non-linear units disposed in several layers where units have specific connections and weights for them. They learn to perform particular tasks through training during which the strength of the connections between units is learned, for this purpose using a cost function that estimates the error of the model and trying to minimize it. In this regard, some artificial networks have been used to replicate animal behaviors and thus being able to link artificial representations to biological ones. However, the real correspondence between the brain and such artificial settings is a contentious issue, given that most of these networks lack of biological realism (Cichy and Kaiser, 2019).

All models for numerical cognition come roughly to the same results, namely, the ability of artificial neural networks to simulate human and non-human animals' behaviors in numerical judgments, i.e., the discrimination of one numerosity from another (Rapp et al., 2020), comparison tasks for relationships of sameness (Nasr et al., 2019), or inequality (Testolin et al., 2020a). However, their relevant implications and proposals regarding the implementation of numerical ability rely on their architectures and main functional properties.

First among all, the model of Meck and Churck proposed a counting mechanism where numerosity and time are encoded by the same magnitudes (Meck and Church, 1983). At the core of their idea, there is the concept of an accumulator that sums the sensory quantity—expressed through sequential impulses or a continuous impulse—which is isomorphic to the magnitude represented. The value of the accumulator is stored as working memory and then compared to another numerosity.

Dehane and Changeaux also employed a summation representation of numerosity in their model (Dehaene and Changeux, 1993). Their functional system consisted of a deep network with four layers. The visual object numerosity is encoded as an ensemble of local Gaussian distributions of activation, which are firstly localized and normalized for size. The information then projects to 15 “summation clusters,” each of which is activated if the overall activity exceeds a certain and different threshold. For every summery summation cluster, there is a final “numerosity cluster,” which is activated only when the preferred numerosity is encoded in the summation cluster. Thus, in this case summation units are just precursors of number-selective units. Even if the model has a very simplified view of relevant biological mechanisms involved and handle only one to five numerosities, nevertheless with only 530 neuronal units it is able to discriminate and account for the behavioral typical signatures of ANS, namely, size and distance effects.

Also, unsupervised models can bring spontaneously to the same numerosity detectors, as demonstrated by another model employing, a very similar architecture and summation-coding principle (Verguts and Fias, 2004). Besides the similar results, this time the network was trained with a backpropagation algorithm for mapping the summation units of the hidden layer to the output layer. Therefore, number-selective neurons can be learned.

Additionally, hierarchical convolutional neural networks have been recruited as models to study sensitivity to numerosity (Nasr et al., 2019). These deep neural networks have been particularly used in computer vision (Fukushima and Miyake, 1982; Krizhevsky et al., 2017), and their architecture is inspired to the visual cortex. They consist of thousands of non-linear units disposed in several layers with sparse connections. Units have specific weights representing specific filters that are applied to the image one after another, in this way modifying the image in order for the network to classify it. Importantly, these weights are autonomously learned by the network during training. In the study on numerosity, the network was trained on 1,2 million images from 1000 categories. Then, it was provided with images representing dot patterns of different numerosities (1–30) and found that 9.6% of the final layer of the feature extraction network units was selective for numerosities. Each of these numerosity-selective neurons was tuned to a preferred numerosity among the 30 presented, and showed approximate tuning with decreased response when deviating from it and a better fit on a logarithmically compressed number line. Moreover, this study suggests the non-necessity of summation units that monotonically modulate—increasing or decreasing—responses with increasing numerosities, due to the the exiguous presence of these types of number coding in the network and the non-necessity of it for the overall conduct of the network.

Another model demonstrated that information about numerosity does not need to be made explicit and does not require specific training. It can emerge as a property of visual stimuli which is spontaneously coded by hidden neurons of deep neural networks (Stoianov and Zorzi, 2012). Indeed, generative neural networks—which are a type of artificial network which aims to reconstruct sensory inputs instead of classifying them—have been used to study the distributed internal representation generated by binary images containing a number of objects up to 32. After training it with 51,200 images, some units were found to be selective for numerosity, others with size. Importantly, these neurons were spatially selective and monotonically represented numerosity, thus resembling results from the parietal cortex in other studies in animals (Roitman et al., 2007). On the contrary, this evidence pushes toward a summation code of numerosity while avoiding the necessity of “number detecting neurons,” since selectivity for numerosity does not necessarily require those properties.

As already mentioned, although the two hypotheses about summation coding and number-selective neurons are not exclusive and could characterize different neuronal populations, there is evidence that suggests summation coding as a more general mechanism (Chen and Verguts, 2013; Zorzi and Testolin, 2018), capable of explaining the properties of the neurons described in monkeys without numerical learning (Viswanathan and Nieder, 2015). A possibility is that number-selective neurons are instead an outcome of training.

Furthermore, basic visuospatial processing in a hierarchical structure together with statistical properties of the object may be sufficient for the emergence of numerical competence even in non-trained networks (Zorzi and Testolin, 2018); moreover, progressive experience can refine and improve this representation resembling developmental advances. It has been suggested that a dedicated system for numerosity also in animals might not be necessary since learning mechanisms of the neural system could be sufficient to extract that information from natural environments, as it is in the case of artificial networks (Testolin et al., 2020b).

These types of neural networks have been also usefully employed in order to disentangle the spiny issue of non-numerical perceptual features that covary with numerosity (Testolin et al., 2020a). Using the stimulus space designed in another study (DeWind et al., 2015), they have been able to statistically estimate the contribute of each non-numerical feature to numerical comparison tasks. They thus showed that whereas both in humans and in the artificial network numerosity is the dominant dimension shaping the decision, the influence of other variables congruent with the magnitude information significantly impacted performance. Numerosity, despite being the primary operator, is not the only one.

All these models that make use of deep neural networks represent different possible solutions to a given problem. Nevertheless, they cannot be considered as true simulations of realistic biological processes, since none of them is actually such. Indeed, despite displaying a great exploratory power for the study on neural processes and demonstrating the feasibility of particular approaches to a problem (Richards et al., 2019), they appear as black boxes (Cichy and Kaiser, 2019) and thereby we should not look for isomorphisms onto which to build explanations. In particular, convolutional neural networks have been shown to be very far from exhibiting a type of classification similar to human one (Szegedy et al., 2014; Goodfellow et al., 2015) and to rely on features that are really obscure to us.

Conversely, other studies employing minimal neural circuits or even single spiking neurons have been able to propose more plausible models of numerical abilities (Vasas and Chittka, 2019; Rapp et al., 2020). Indeed, with minimum training and scarce computational resources, they could simulate sequential visual discrimination of bees and thus demonstrate that even action potentials produced by a single spiking neuron can endow with basic discrimination of numerosity.

## Conclusions

The main goal of this review was to provide a comparative portrait of the neurobiology of numerical cognition. Considering the several possible solutions to the “numerical” computational problem in many organisms, in order to understand the *why* and the *how* underlying it, we must look at the biological implementations. For this reason, we tried to cover the main subpallial and pallial contributes to the approximate representation of quantity, encompassing evidences from organisms of different taxonomic groups. Above all, what emerged is that numerosity might be processed straightforward, without the need to speculate on complex networks or sophisticate brains. However, numerical estimation reveals to be at the crossroad of other cognitive processes—such as attention, decision-making, or memory—which have been shadowed by the great amount of research pointing toward the ability of different organisms to discriminate or not discrete quantities. Therefore, as highlighted by others (Cheyette and Piantadosi, 2019; Vasas and Chittka, 2019), further studies should try to characterize better the strategies involved in numerical tasks in order to disclose important neurobiological features engaged.

## Author Contributions

EL, MP, and GV: conceptualization. GV: resources, writing-review and editing, and funding acquisition. EL and MP: writing-original draft. EL: visualization. All authors: contributed to the article and approved the submitted version.

## Conflict of Interest

The authors declare that the research was conducted in the absence of any commercial or financial relationships that could be construed as a potential conflict of interest.

## Abbreviations

ANS: approximate number system
Dc: dorsocentral division of fish telencephalon
IPS: intraparietal sulcus
LIP: lateral intraparietal cortex
LGN: lateral geniculate nucleus
NCL: nidopallium caudolaterale
OTS: object tracking system
PFC: prefrontal cortex
VIP: ventral intraparietal cortex
V1: primary visual cortex, striate cortex
V2–V3: extrastriate cortices.
