# Supplementary material for: Numerosities and Other Magnitudes in the Brains: A Comparative View
Source: Front Psychol. 2021 Apr 15;12:641994. doi: 10.3389/fpsyg.2021.641994 (PMC8082025; doi:10.3389/fpsyg.2021.641994)
Supplement: Supplementary file 1 [file Table_1.DOCX]

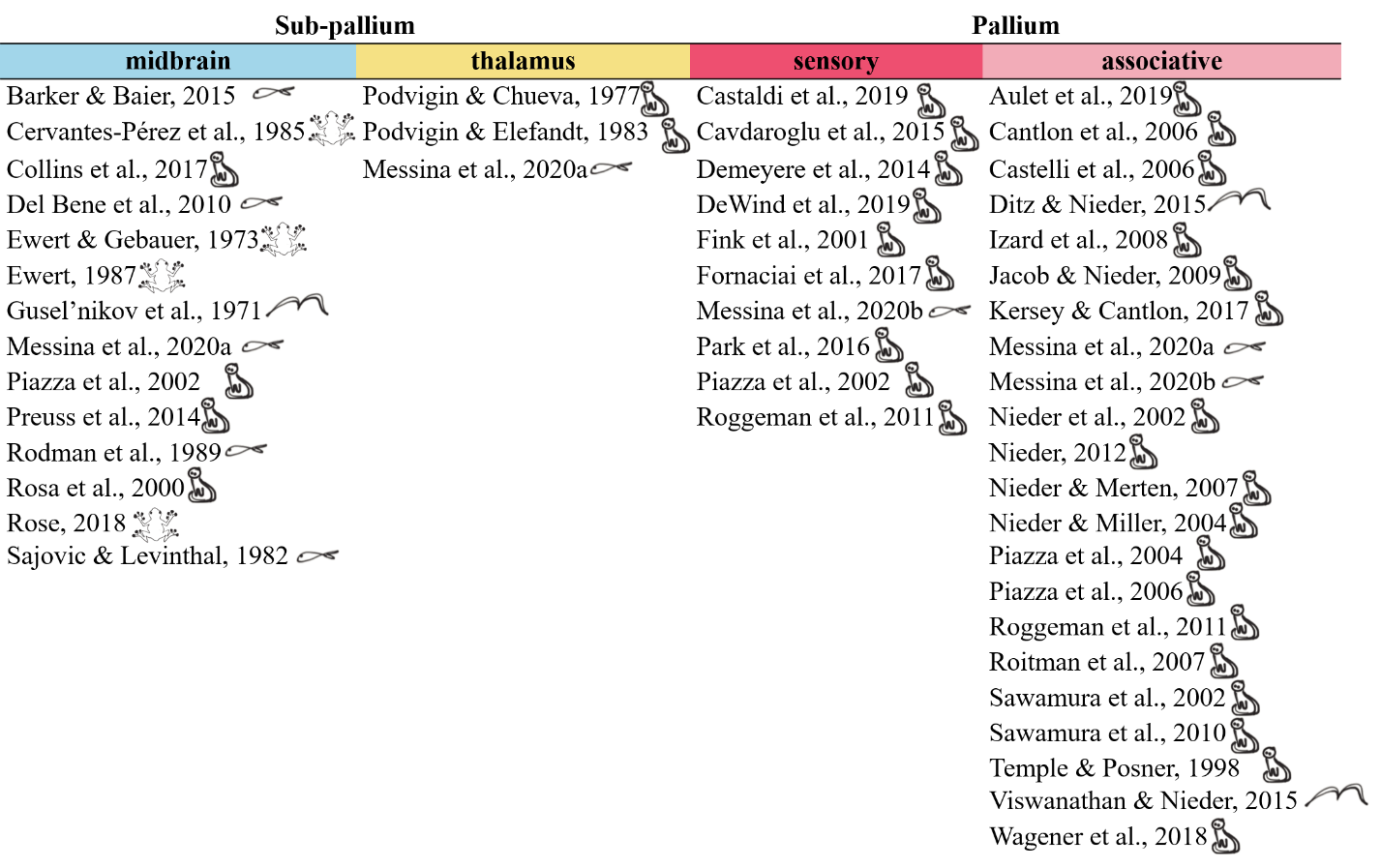


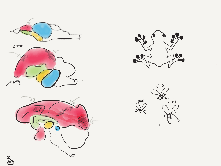

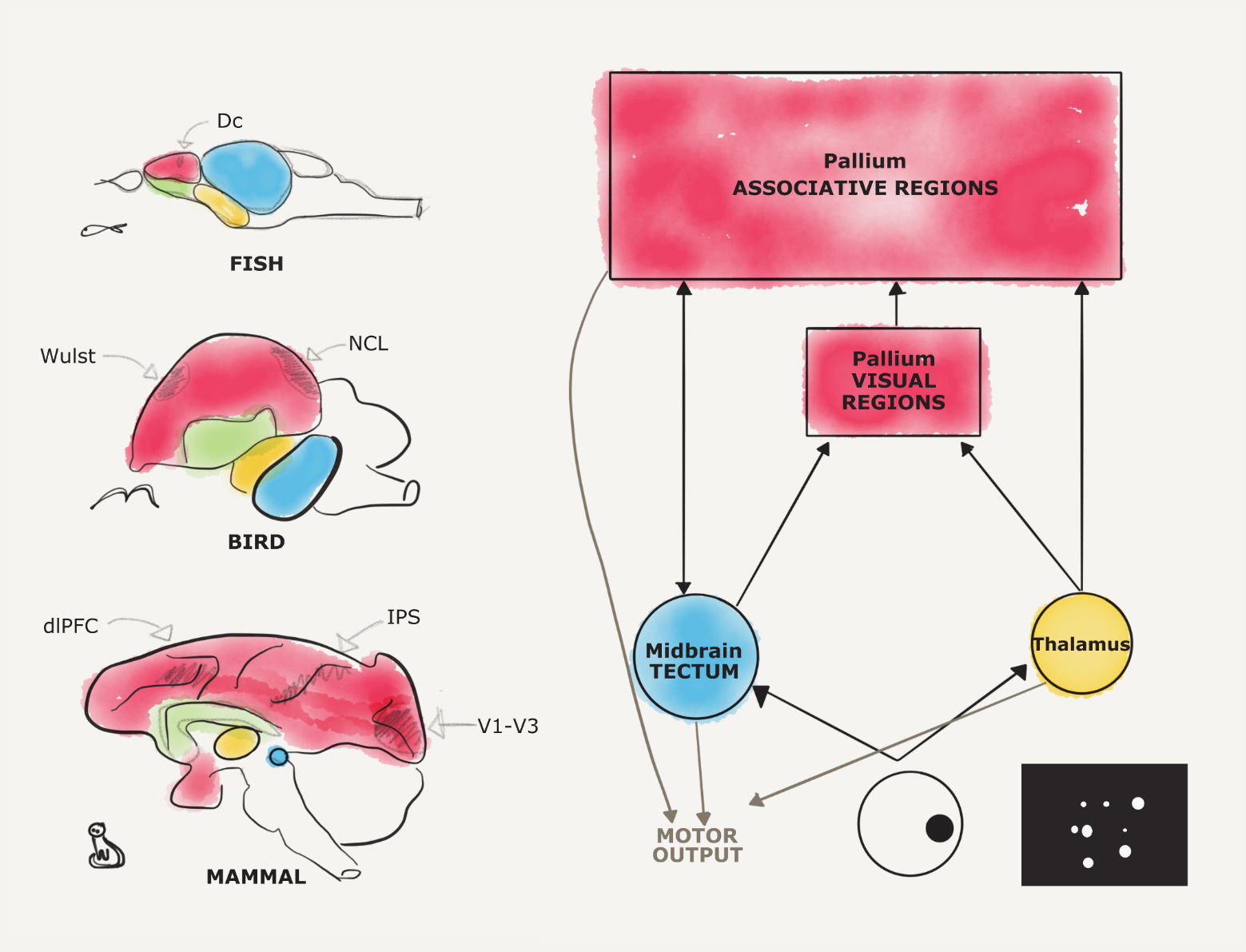

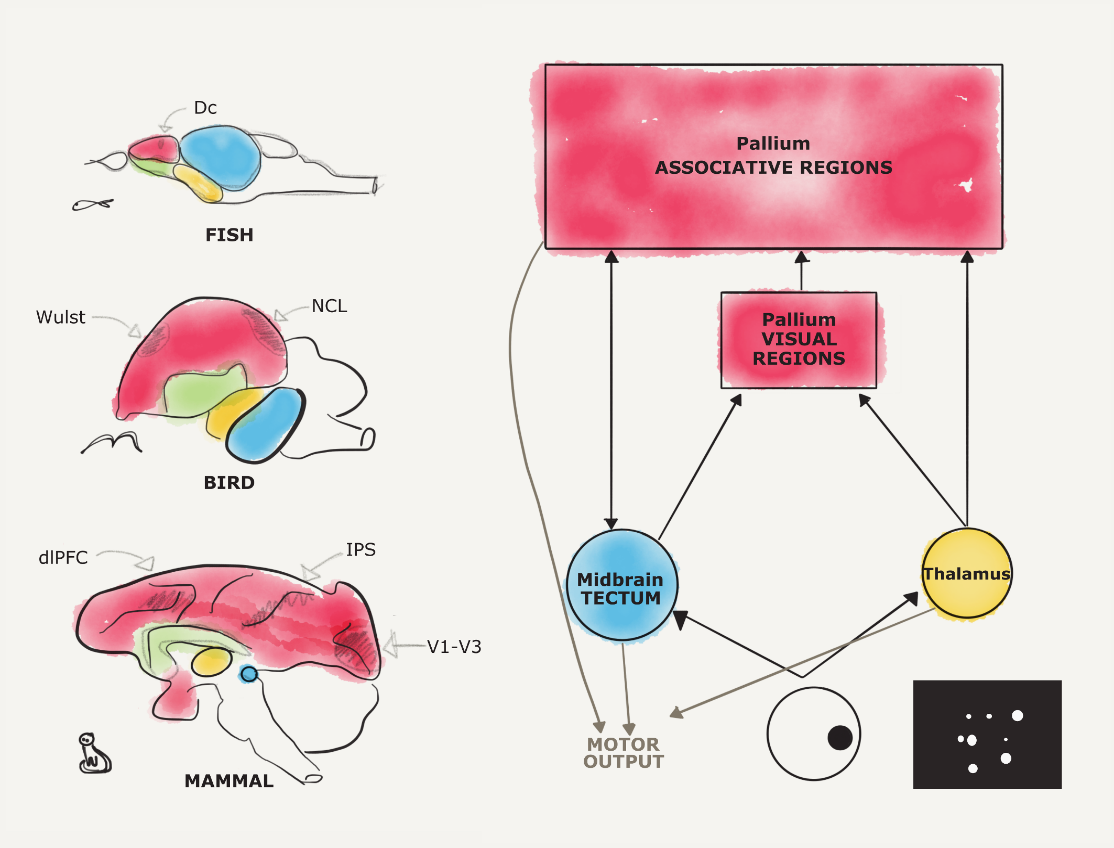

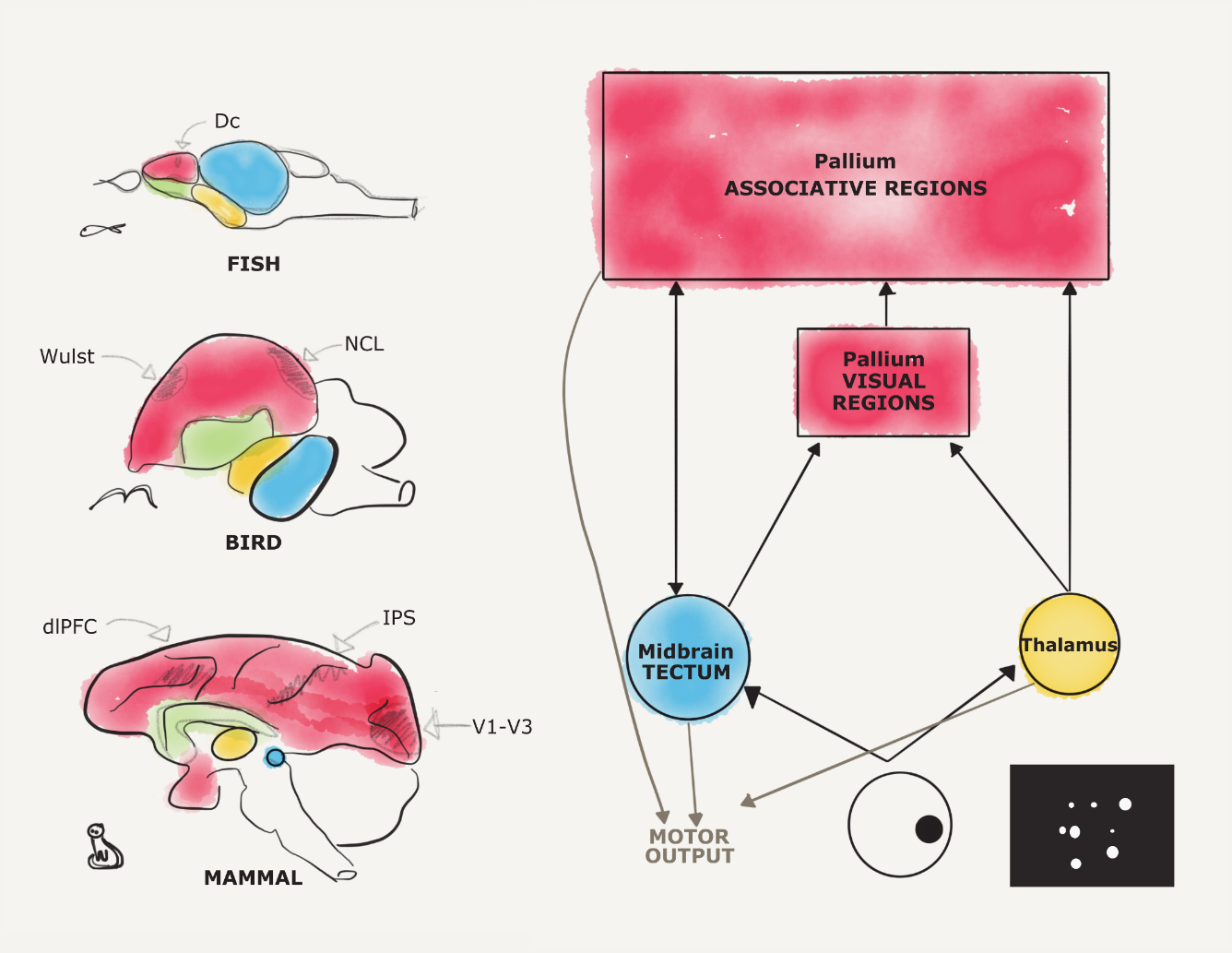
Table 1: Papers reporting evidence for sub-pallial and pallial involvement in magnitude estimation. Sub-pallium and pallium are subdivided respectively into midbrain (blue) and thalamus (yellow), and sensory (red) and associative (pink) regions. A graphic symbol close to each reference indicates the class of vertebrates the study refers to (i.e., mammals , birds , fishes or amphibians ).
